# Supplementary material for: Long-term deep-supercooling of large-volume water and red cell suspensions via surface sealing with immiscible liquids
Source: Nat Commun. 2018 Aug 10;9:3201. doi: 10.1038/s41467-018-05636-0 (PMC6086840; doi:10.1038/s41467-018-05636-0)
Supplement: Supplementary file 1 — Supplementary Information [file 41467_2018_5636_MOESM1_ESM.pdf]

Supplementary Information

Long-term deep-supercooling of large-volume water and red cell suspensions via surface sealing with immiscible liquids

Huang et al.

Supplementary Note 1

Derivation of Young's equation at triple interface

When an ice embryo forms at the air/water interface, water wets ice at a contact angle of θ_{iwa} as shown in Supplementary Figure 1a. The vector components of interface tensions γ^{wi} (between water and ice), γ^{wa} (between water and air), and γ^{ia} (between ice and air) are depicted in the inset. To achieve force balance at x coordinate, following equation has to be satisfied,

$$\gamma^{\mathrm{ia}} = \gamma^{\mathrm{wi}} + \gamma^{\mathrm{wa}}\cos\theta_{\mathrm{iwa}}$$

Or

$$\gamma^{\mathrm{wi}} = \gamma^{\mathrm{ia}} - \gamma^{\mathrm{wa}}\cos\theta_{\mathrm{iwa}}$$

Of note, these equations also can be interpreted as the requirement for minimum free energy in the triple phase system [3]. The contact angle θ_{iwa} generally is not zero (12 ° as measured in [4]). However, when the surface is sealed by immiscible oils, the water contact angle on ice (θ_{iwo}) approaches toward zero to minimize the overall free energy of interfaces [5, 6].

Stochastic process of ice nucleation and freezing

The formation of a critical ice embryo, i.e. a successful nucleation, in metastable supercooled water is generally regarded as a stochastic process that does not depend on the number of previous nucleation trials or correlate to other nucleation events during the same period [7, 8]. In addition, heterogenous nucleation is the major type of crystallization in this study since homogeneous nucleation in water occurs at much lower temperatures (around - 40 °C). As a result, the heterogeneous ice nucleation on water surfaces/interfaces would follow Poisson statistics

$$\mathrm{In}\left(1 - f_{\mathrm{f}}(t)\right) = -J(T)\cdot S\cdot t$$

where $f_{\mathrm{f}}(t)$ is the freezing frequency after supercooling of a period t, $J(T)$ is the nucleation rate at temperature T, and S is the area of heterogeneous nucleation sites. Therefore, for water samples of the same volume and shape under a constant temperature, the non-frozen (supercooled) fraction is expected to decline exponentially with time.

However, in our experiments we found that $f_{\mathrm{f}}(t)$ of DSC water with oil sealing does not change significantly after Day 3 as shown in Fig. 1, Fig. 3, Supplementary Figure 2, and Supplementary Figure 3. These results indicate that the heterogeneous ice nucleation in DSC water sealed by oil phase does not follow the conventional theory of stochastic nucleation processes at the interface. Particularly, since 45.8% of 100 ml DSC water (-16 °C) sealed by PO are frozen post 1-day storage (Fig. 1d), $\mathrm{In}\,(1-0.458) = -J(-16\,^{\circ}\mathrm{C})\cdot S\cdot 1$, which gives rise to $J(-16\,^{\circ}\mathrm{C})\cdot S$ = 0.612. Therefore, the expected fraction of unfrozen samples, $1 - f_{\mathrm{f}}(t) = e^{-0.612t}$, would decrease exponentially with storage time for DSC water of the same volume and shape at -16 °C. As a result, the fraction of unfrozen samples would be 0.22% and 2.5×10^{-25} % on Day-10 and Day-100, respectively, which implies almost all the sample would be frozen after 10-day storage. However, we observed that 22.9% (8 out of 35) of samples were still unfrozen post 100-day storage in our experiments. Moreover, no freezing event occurred between Day-3 and Day-100; that is all the samples that were unfrozen on Day-3 remained unfrozen till the end of our

experiments on Day-100. These observations strongly demonstrated a non-stochastic process of ice nucleation in our DSC water-oil phase systems.

Using similar heuristics, our experimental results also suggest that the freezing we observed is not due to homogeneous ice nucleation either, where the formation of critical ice embryo is caused by spontaneous aggregation of water molecules via random translational, rotational, and vibrational movements that would conform stochastic process [9]. The exact kinetics and statistics of this heterogeneous ice nucleation in DSC water sealed by oil phase is still unknown and detailed future investigations are certainly warranted.

Freezing point depression due to oil-water mixing

When a water sample is sealed by an oil phase (i.e mixed oils, and pure alkanes and alcohols) for DSC, the "immiscible" oil might slightly dissolve in supercooled water to decrease the equilibrium melting temperature T_e below 0 °C, the equilibrium melting point of pure water under atmospheric conditions. We, therefore, quantified the potential depression of freezing point due to this effect and assessed whether it's comparable to the high degree of supercooling we observed in our experiments. According to the Bladgen's Law, the extent of freezing point depression ΔT_{F} can be calculated by

$$\Delta T_{\mathrm{F}} = ibK_{\mathrm{F}}$$

where i is the Van't Hoff factor (i = 1 for nonelectrolytes or oil phase in this study), b is the molality of oil phase in water, and K_{F} is the cryoscopic constant (K_{F} = 1.85 K kg mol^-1 for water). Therefore, ΔT_{F} can be determined by the solubility of sealing oils in water at DSC temperatures. Solubility of oils in metastable water at DSC temperatures are not readily available; as such we have assessed ΔT_{F} using the available solubility data of oils in water under room temperature. This approach, likely, leads to an overestimation since solubility of oils in water typically increases with temperature [10].

For oil mixtures (MO, OO, PO, NO, and PDMS) and linear alkanes (C_5 ~ C_{11}) utilized in this study, the maximum solubility is 0.04 g L^-1 (or 0.55 mM) (C_5 in water), and the corresponding estimate for ΔT_{F} is less than 1.03×10^{-3} °C, which is negligible compared to the degree of supercooling ΔT (10 to 20 °C) achieved using these oil phases as sealing agents.

For alcohols used in this study, the maximum solubility is 73 g L^-1 (or 0.98 M) (C_4OH in water at room temperature) and the corresponding estimate for ΔT_F is less than 1.82 °C. This likely overestimated freezing point depression accounts for about 9.1% of ΔT (20 °C) enabled by alcohol sealing. Moreover, the DSC water and sealing alcohols are likely not mixed altogether. The stable contact interface with strong hydrogen bonding on the head and a long hydrophobic tail of alcohols, low molecular mobility, and viscous water at - 20 °C would significantly impede the diffusion of alcohol molecules into water. We, therefore, conclude that the depression of freezing temperature due to oil-water mixing does not play a significant role in achieving the observed high degree of supercooling in our experiments.

Supplementary Data

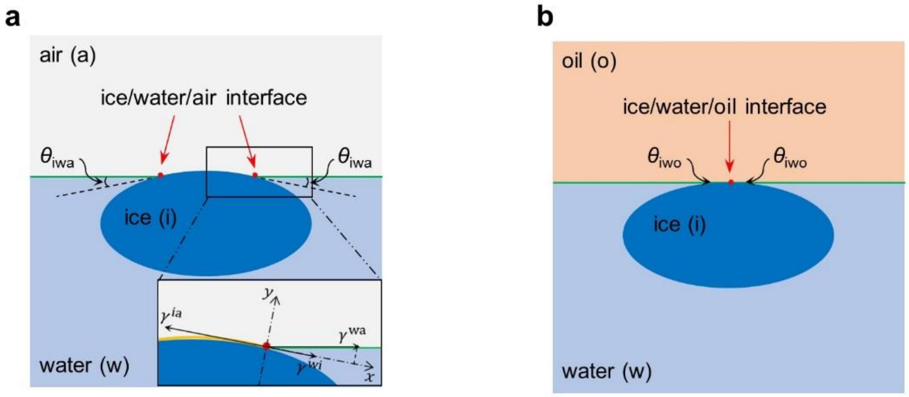

Supplementary Figure 1. Schematics for ice/water/air **a** and ice/water/oil **b** contacts. θ_{iwa} and θ_{iwo} are water contact angles on ice/water/air and ice/water/oil interfaces, respectively. In the inset of **a**, symbols γ^{wa}, γ^{wi}, and γ^{ia} are the interfacial tensions for water/air, water/ice, and ice/air interfaces, respectively

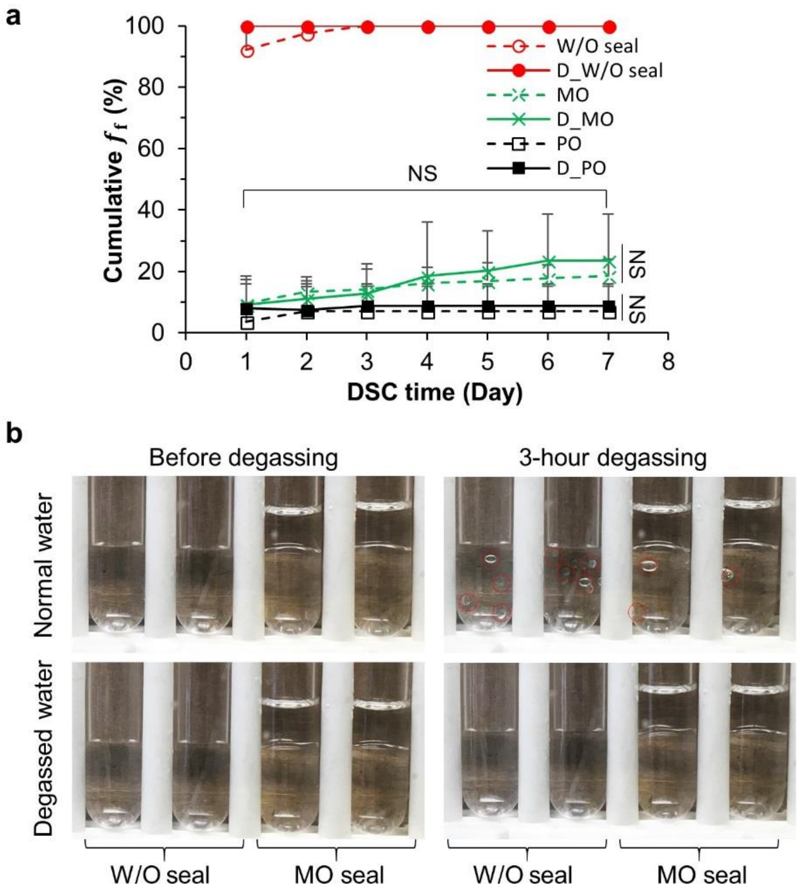

Supplementary Figure 2. Freezing comparison between normal and degassed DSC water. **a** Cumulative freezing frequencies of normal and degassed DSC water at - 16 °C over 7 days. “D_W/O seal”, “D_MO”, and “D_PO” represent degassed water without sealing, surface sealing with MO, and PO, respectively. n = 6, N = 60, NS: $p > 0.05$. Error bars represent standard deviations. **b** Degassing images for normal and degassed water with or without MO sealing. The red dash circles indicate air bubbles precipitated under vacuum.

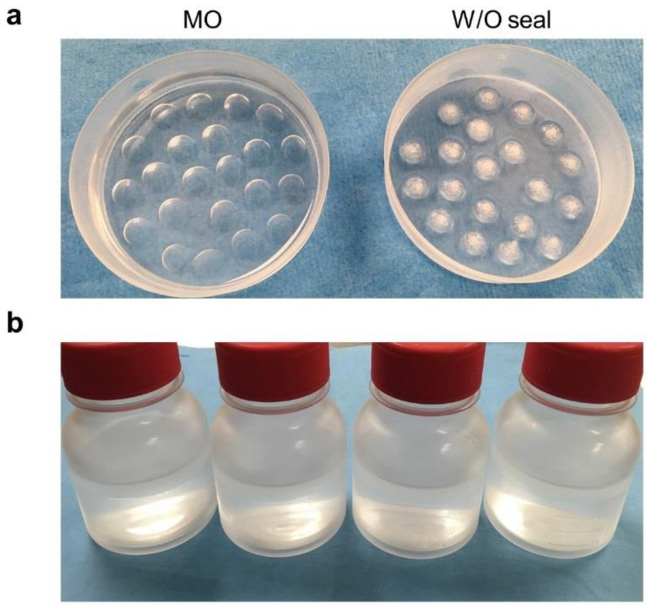

Supplementary Figure 3. Representative images of DSC water of various volumes. **a** 30 μl droplets with or without MO sealing post 1-day DSC at - 16 °C. **b** 100 ml deionized (DI) water sealed by 16 ml PO post 100-day DSC at - 16 °C. All possible freezing events occur before Day-3, and 8 out of total 35 (n = 7, N = 35) bottles of water maintain unfrozen after 100-day storage.

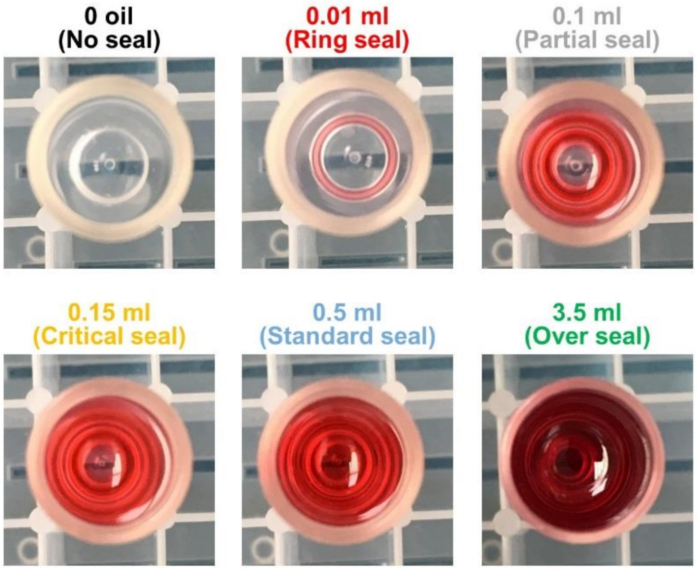

Supplementary Figure 4. Vertical view of water surface in round-bottomed tubes sealed by MO of various volumes. The oil was stained by Oil Red O for enhanced contrast. Newton's rings were observed due to the curvature of sealing oil near the tube wall.

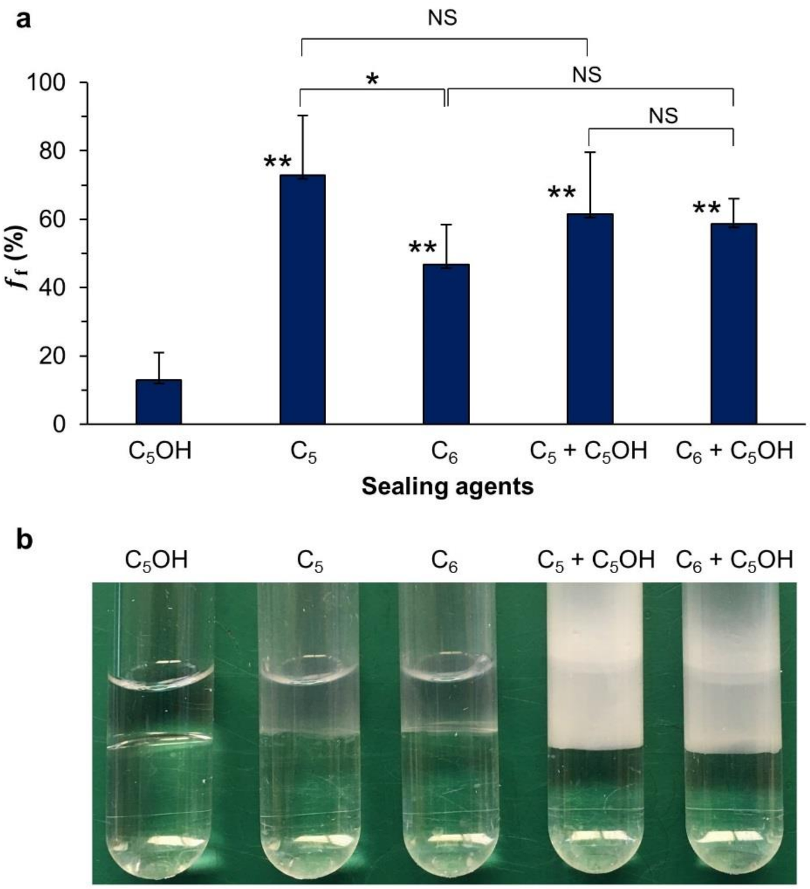

Supplementary Figure 5. Freezing frequencies of DSC water sealed by binary combinations of alcohol and alkane. 1 ml DSC water at -20 °C was sealed with 0.5 ml alcohol (C_5OH), alkanes (C_5 and C_6), and their binary combinations (C_5 + C_5OH and C_6 + C_5OH). In the binary mixtures, the concentration of the alcohol (C_5OH) is 1% (v/v). **a** f_f of DSC water post 1-day supercooling. The marks just above the columns (**) indicate the *p* value between C_5OH and respective sealing agent. n = 7, N = 87. Error bars represent standard deviations. **b** Corresponding images of water loaded with these sealing agents before supercooling test. Significant portion of water is sucked into the binary sealing agents (C_5 + C_5OH and C_6 + C_5OH).

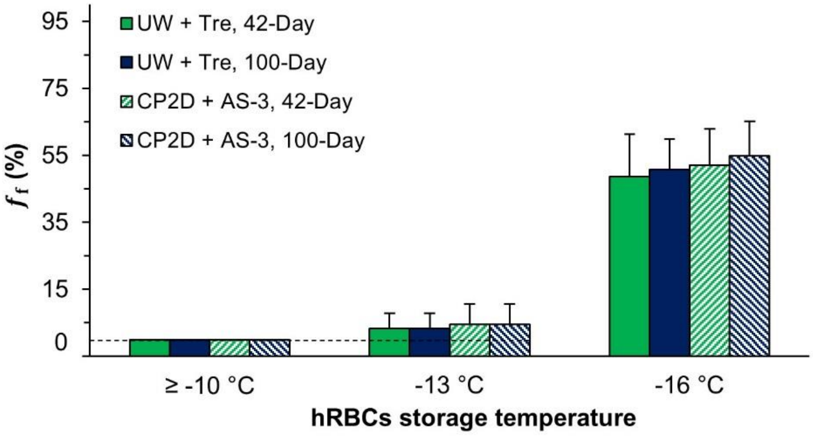

Supplementary Figure 6. Freezing frequencies of 1 ml DSC hRBCs suspensions sealed with 0.5 ml PO. 10 million hRBCs were suspended in different storage solutions (UW + Tre and CP2D + AS-3), stored under different temperatures (4, -7, -10, -13, and -13 °C) for 42 and 100 days. n = 5, N = 56. Error bars represent standard deviations.

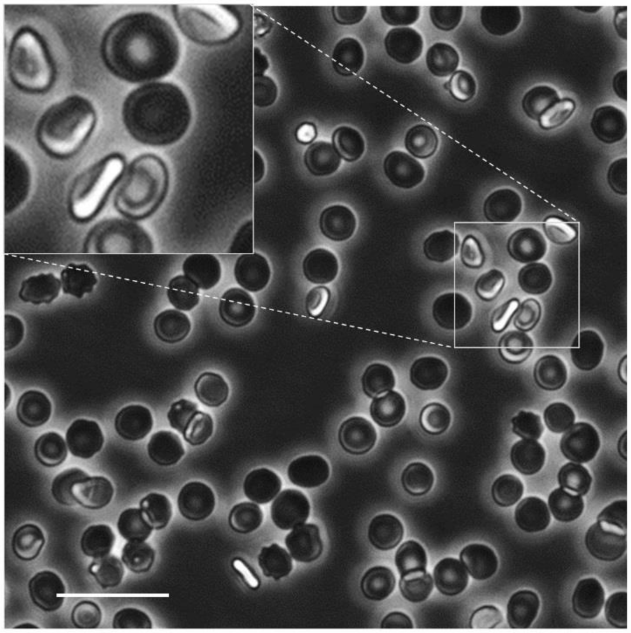

Supplementary Figure 7. Typical phase micrographs of fresh hRBCs in phosphate-buffered saline (PBS). Scale bar represents 50 µm.

Supplementary References

1. Wians Jr FH, Miller CL, Heald JI, Clark H. Evaluation of a direct spectrophotometric procedure for quantitating plasma hemoglobin. *Laboratory Medicine* **19**, 151-155 (1988).

2. Noe DA, Weedn V, Bell WR. Direct spectrophotometry of serum hemoglobin: an Allen correction compared with a three-wavelength polychromatic analysis. *Clinical chemistry* **30**, 627-630 (1984).

3. Makkonen L. Young's equation revisited. *Journal of Physics: Condensed Matter* **28**, 135001 (2016).

4. Knight CA. The contact angle of water on ice. *Journal of Colloid and Interface Science* **25**, 280-284 (1967).

5. Tabazadeh A, Djikaev YS, Reiss H. Surface crystallization of supercooled water in clouds. *Proc Natl Acad Sci U S A* **99**, 15873-15878 (2002).

6. Konno A, Izumiyama K. On the relationship of the oil/water interfacial tension and the spread of oil slick under ice cover. In: *Proceedings of the 17th International Symposium on Okhotsk Sea and Sea Ice* (2002).

7. Koop T, Luo B, Biermann UM, Crutzen PJ, Peter T. Freezing of HNO3/H2SO4/H2O solutions at stratospheric temperatures: Nucleation statistics and experiments. *The Journal of Physical Chemistry A* **101**, 1117-1133 (1997).

8. Niedermeier D*, et al.* Heterogeneous ice nucleation: exploring the transition from stochastic to singular freezing behavior. *Atmospheric Chemistry and Physics* **11**, 8767-8775 (2011).

9. Krämer B*, et al.* Homogeneous nucleation rates of supercooled water measured in single levitated microdroplets. *The Journal of Chemical Physics* **111**, 6521-6527 (1999).

10. Paasimaa S. Factors affecting water solubility in oils. *Vaisala News* **169**, 24-25 (2005).
