# Supplementary material for: Long-term deep-supercooling of large-volume water and red cell suspensions via surface sealing with immiscible liquids
Source: Nat Commun. 2018 Aug 10;9:3201. doi: 10.1038/s41467-018-05636-0 (PMC6086840; doi:10.1038/s41467-018-05636-0)
Supplement: Supplementary file 3 — Description of Additional Supplementary Files [file 41467_2018_5636_MOESM3_ESM.pdf]

Description of Additional Supplementary Files

File Name: Supplementary Movie 1
Description: Ultrasonication for 1 ml DSC water sealed by MO. The water is supercooled at - 20 °C for 1 day and the ultrasonic frequency is 40 kHz.

File Name: Supplementary Movie 2
Description: Ultrasonication for 1 ml DSC water sealed by undecane (C11). The water is supercooled at - 20 °C for 1 day and the ultrasonic frequency is 40 kHz.

File Name: Supplementary Movie 3
Description: Ultrasonication for 1 ml DSC water sealed by 1-butanol (C4OH). The water is supercooled at - 20 °C for 1 day and the ultrasonic frequency is 40 kHz.
